# Curli Functional Amyloid Systems Are Phylogenetically Widespread and Display Large Diversity in Operon and Protein Structure

**DOI:** 10.1371/journal.pone.0051274

**Published:** 2012-12-12

**Authors:** Morten S. Dueholm, Mads Albertsen, Daniel Otzen, Per Halkjær Nielsen

**Affiliations:** 1 Department of Biotechnology, Chemistry, and Environmental Engineering, Aalborg University, Aalborg, Denmark; 2 Interdisciplinary Nanoscience Center (iNANO), Centre for Insoluble Protein Structures (inSPIN), Department of Molecular Biology and Genetics, Aarhus University, Aarhus, Denmark; University of Birmingham, United Kingdom

## Abstract

*Escherichia coli* and a few other members of the Enterobacteriales can produce functional amyloids known as curli. These extracellular fibrils are involved in biofilm formation and studies have shown that they may act as virulence factors during infections. It is not known whether curli fibrils are restricted to the Enterobacteriales or if they are phylogenetically widespread. The growing number of genome-sequenced bacteria spanning many phylogenetic groups allows a reliable bioinformatic investigation of the phylogenetic diversity of the curli system. Here we show that the curli system is phylogenetically much more widespread than initially assumed, spanning at least four phyla. Curli fibrils may consequently be encountered frequently in environmental as well as pathogenic biofilms, which was supported by identification of curli genes in public metagenomes from a diverse range of habitats. Identification and comparison of curli subunit (CsgA/B) homologs show that these proteins allow a high degree of freedom in their primary protein structure, although a modular structure of tightly spaced repeat regions containing conserved glutamine, asparagine and glycine residues has to be preserved. In addition, a high degree of variability within the operon structure of curli subunits between bacterial taxa suggests that the curli fibrils might have evolved to fulfill specific functions. Variations in the genetic organization of curli genes are also seen among different bacterial genera. This suggests that some genera may utilize alternative regulatory pathways for curli expression. Comparison of phylogenetic trees of Csg proteins and the 16S rRNA genes of the corresponding bacteria showed remarkably similar overall topography, suggesting that horizontal gene transfer is a minor player in the spreading of the curli system.

## Introduction

Non-pathogenic as well as human and animal pathogenic *Escherichia coli* isolates and *Salmonella enterica* serovars are able to produce functional bacterial amyloids (FuBA) collectively referred to as curli [1]–[3]. FuBA are defined as bacterial protein polymers with a fibrillar structure in which the protein monomers fold as β-sheets stacked perpendicular to the fibril axis [4], [5].

Curli fibrils are involved in bacterial attachment to surfaces, cell aggregation and are an important part of the extracellular matrix required for the formation of mature biofilms [6]–[9]. Curli fibrils are also considered important virulence factors as they interact with a wide range of host proteins, which are proposed to facilitate bacterial dissemination through the host. These include extracellular matrix proteins [10]–[12] and contact-phase proteins [13]–[16]. Curli are recognized by Toll-like receptors, leading to the activation of the innate immune system [17], [18]. Curli are therefore considered pathogen-associated molecular patterns (PAMPs).

A highly regulated pathway involving two divergently expressed operons is required for curli biogenesis. The *csgBAC* operon encodes the major curli subunit, CsgA, and its homolog CsgB, which after translocation to the cell surface acts as a nucleus for polymerization of soluble CsgA [3], [19]. It also encodes CsgC, which is required for correct assembly of the mature curli fimbriae [20], [21]. The *csgDEFG* operon encodes CsgD, a transcriptional activator of the *csgBAC* operon, together with CsgE and CsgF, which act as chaperones and are required for effective curli assembly. Finally, it encodes CsgG, a helical outer-membrane macromolecular exporter, important for secretion of the curli subunits through the outer-membrane [3], [19], [20], [22]–[24], which works in concert with CsgE to facilitate CsgA secretion [25].

Curli homologs have previously been identified within some, but far from all genera of the Enterobacteriales using either purification (*Escherichia* and *Salmonella*) or PCR-based methods targeting the *csgA* or *csgD* genes (*Escherichia/Shigella*, *Salmonella*, *Citrobacter* and *Enterobacter*) [12], [26], [27]. Curli fibrils may, however, be phylogenetically much more widespread and could consequently be important virulence factors for other pathogens.

The growing number of genome-sequenced bacteria spanning many phylogenetic taxa allows a reliable determination of the phylogenetic and structural diversity of genes and related proteins by bioinformatic tools [28], [29].

In this study, homologs of the curli associated Csg proteins were found to be phylogenetically much more widespread than initially assumed, spanning at least four bacterial phyla. A high degree of variability within the operon structure between bacterial taxa suggests that the curli fibrils might have evolved to fulfill specific functions and may consequently be encountered frequently in environmental as well as pathogenic biofilms.

## Results

### Curli Genes are Phylogenetically Widespread

Homologous curli systems were initially identified within the refseq protein database using PSI-Blast searches with Csg proteins from *E. coli* str. K-12 substr. MG1655 as query sequences. The hits were manually curated and additional homologs were identified by examination of the genetic neighborhood of the hits. The identified Csg homologs showed a high degree of variability in terms of primary structure, and sequence identity of the CsgA homologs were only 9–33% for the non-Enterobacteriales compared to CsgA from the *E. coli* strain.

Purely sequence-based methods, such as Blast searches, may not be able to detect evolutionarily related protein sequences if these have been subjected to intensive recombination and fast evolution [30]. The low sequence identity between Csg homologs of phylogenetically closely related species suggests that divergence could provide a problem when searching for curli homologs.

Profile hidden Markov models (HMMs) are much better at detecting remote homology between proteins than Blast searches [30]. The curated Csg homologs were therefore used to generate HMMs of the Csg proteins. As CsgA and CsgB are internal homologs and these proteins are highly variable, a combined CsgA/B HMM were constructed based solely on the curli repeat regions situated as described by Barnhart and Chapman [31]. The HMM models were able to identify additional curli homologs. The additional hits were curated as described earlier and the expanded Csg protein database were used to generate improved HMM models (Table 1). The iterative process was repeated until no additional homologs could be identified.

**Table 1:** Validation of the Hidden Markov Models and Comparison to Current Pfam-B Models.

Target	Model	Hits	Correct hits	Missing hits
CsgA/B (curli repeat)	Pfam (PF07012)^1^	354	354/354 (100%)	47/401 (12%)
CsgA/B (curli repeat)	This study	379	374/379 (99%)	27/401 (7%)
CsgC	This study	105	104/105 (99%)	1/105 (1%)
CsgD	This study	16948	135/16948 (1%)	1/136 (1%)
CsgE	Pfam (PF10627)	161	159/161 (99%)	1/160 (1%)
CsgE	This study	159	159/159 (100%)	1/160 (1%)
CsgF	Pfam (PF10614)	172	172/172 (100%)	0/172 (0%)
CsgF	This study	172	172/172 (100%)	0/172 (0%)
CsgG	Pfam (PF03783)	601	171/601 (26%)	2/173 (1%)
CsgG	This study	266	171/266 (59%)	2/173 (1%)
CsgH	This study	25	23/25 (92%)	0/23 (0%)

1 The size and location of the curli repeat region assigned by this model does not agree with the curli repeat regions described by [31].

The curated HMMs in general performed very well (Table 1). The CsgA/B repeat model was able to detect 93% of all CsgA/B proteins in our homolog database. The remaining 7% were CsgA/B proteins, which contained no or only one repeat region. These proteins are therefore assumed to be non-functional. The few hits on non-curli proteins represented four very large *Shewanella* adhesion proteins, which interestingly contained regularly spaced curli-like repeats as well as a single archaeal protein, which also had regularly, spaced curli-like repeats. The curated CsgA/B repeat model was able to detect more CsgA/B homologs than the curlin repeat Pfam model (PF7012), although the latter did not hit false positives (Table 1). However, a big advantage of the curated model is that it is able to correctly assign the location of the repeat regions in line with those described by Bernhart and Chapman [31]. The curated CsgC, CsgE, CsgF and CsgH models were all very sensitive and highly specific. The CsgE model performed better that the current Pfam-B model (PF10627), whereas the CsgF model had a similar performance as Pfam-B model (PF10614). The curated CsgG HMM was much more specific than the current Pfam model (PF3783), although it still included 41% false positives. The non-specific CsgG hits are likely paralogs, and indicate that CsgG is part of a larger protein family. The CsgD HMM has a good sensitivity, but cannot be used to probe curli systems as this protein is part of a very large protein family.

**Figure 1:**
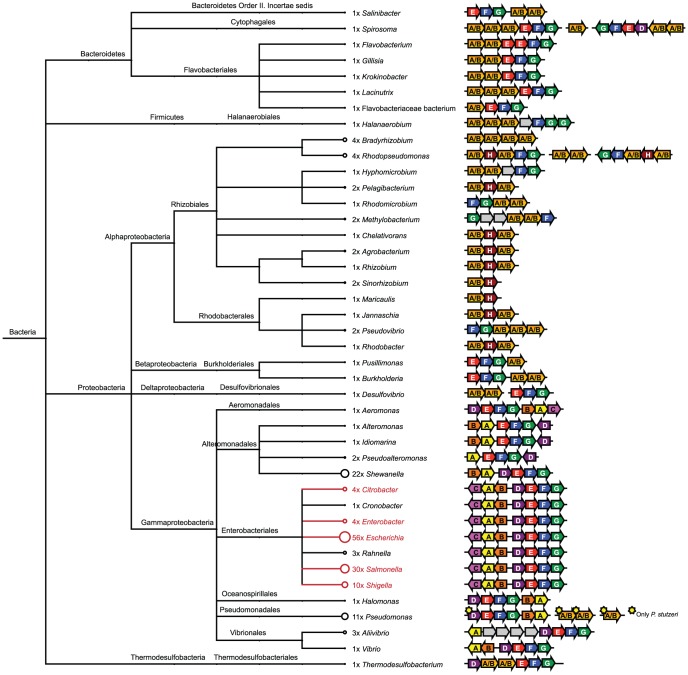
Phylogenetic Distribution of the Curli Systems and Operon Structure. Taxonomic analysis was performed based on the NCBI taxonomy and visualized using MEGAN [49]. The number of strains containing curli systems within each genus is indicated next to the taxonomic units. Note that these numbers are highly influenced by the number of sequenced strains within each phylogenetic group and therefore do not reflect the prevalence of curli systems within these groups. Genera highlighted in red represent genera where curli systems have been previously described. Organization of the curli operons is illustrated for each genus.

The combination of HMM searches and manual examination of the surrounding gene neighborhoods allowed identification of all previously known curli systems. These, however, represent only a tiny part of the phylogenetic curli diversity (Figure 1 and Table S1). The majority of the curli systems were found within the Proteobacteria. Surprisingly, several curli systems were also found within Bacteroidetes and within a single Firmicutes and Thermodesulfobacteria strain. Within the Proteobacteria most curli systems were found within the Alpha- and Gammaproteobacteria, although two systems were found in the Betaproteobacteria, both within the order Burkholderiales, and a single system were found within the order Desulfovibionales of the Deltaproteobacteria. This shows that the genes coding for curli systems are phylogenetically much more widespread than previously appreciated. It should be noted that only some of the families within the previously mentioned phyla contain curli homologs. Csg homologs are for example not present within any of the genome sequenced *Klebsiella* and *Yersinia* strains, although these bacteria are both members of the Enterobacteriales and phylogenetically closely related to *E. coli*.

### Conservation and Organization of *csg* Genes

Conservation of curli genes shows some interesting variations among bacterial classes (Figure 1). Homologs to all *csg* genes can be found within every orders of the Gammaproteobacteria with the exception of *csgC*, which is only found within the Enterobacteriales and Aeromonadales. The Delta- and Betaproteobacteria lack homologs to *csgC* and *csgD*, while the Alphaproteobacteria all lack *csgC*, *csgD* and *csgE*. In addition, approximately half of the genera within the Alphaproteobacteria also lack *csgE* and *csgF*. It is interesting to notice that many of the Alphaproteobacteria carry an additional gene (*csgH*), which is always situated next to *csgA/B* and does not show similarity to any other genes in the refseq database. This gene might fulfill the role of the missing *csgE* and *csgF* genes. The single Firmicutes lacks *csgC*, *csgD* and *csgE*, whereas the lone Thermosulfobacteria only lacks *csgC*. Finally, all genera of the Bacteroidetes lack *csgC* and *csgD*, with the exception of *Spirosoma*, which have a *csgD* homolog. It is only possible to distinguish CsgA and CsgB homologs within the Gammaproteobacteria. Interestingly, members of some genera contain up to six *csgA/B* homologs, as is the case for *Spirosoma* and *Rhodopseudomonas*. These additional copies are not similar and might be used to modulate the physiochemical properties of the final amyloid fibrils. This is supported by the fact that the additional copies are often situated in additional curli operons and may therefore be regulated independently of the main curli operon.

An examination of the genetic organization of the homologous *csg* genes within the Gammaproteobacteria shows a high degree of reorganization compared to the strict organization known for members of the Enterobacteriales (Figure 1). The organization of the *csg* genes into the divergently expressed *csgDEFG* and *csgBAC*, described for *E. coli* and *Salmonella*, is only seen within the Enterobacteriales and the Vibrionales. The two operons are conserved in the Aeromonadales and Oceanospirillales, but here they are oriented in the same direction. In the Pseudomonadales and the *Shewanella* genus of the Alteromonadales the operons are divergently oriented, but the *csgD* homologs are separated from the *csgDEFG* operon and seem to be transcribed alone. Within *Alteromonas* and *Pseudoalteromonas* of the Alteromonadales all *csg* genes, apart from *csgD*, form a single operon. *csgD* is divergently oriented and located downstream the *csgBAEFG* operon.

### CsgA Homologs are Highly Variable whereas CsgB Structure is Conserved

**Figure 2:**
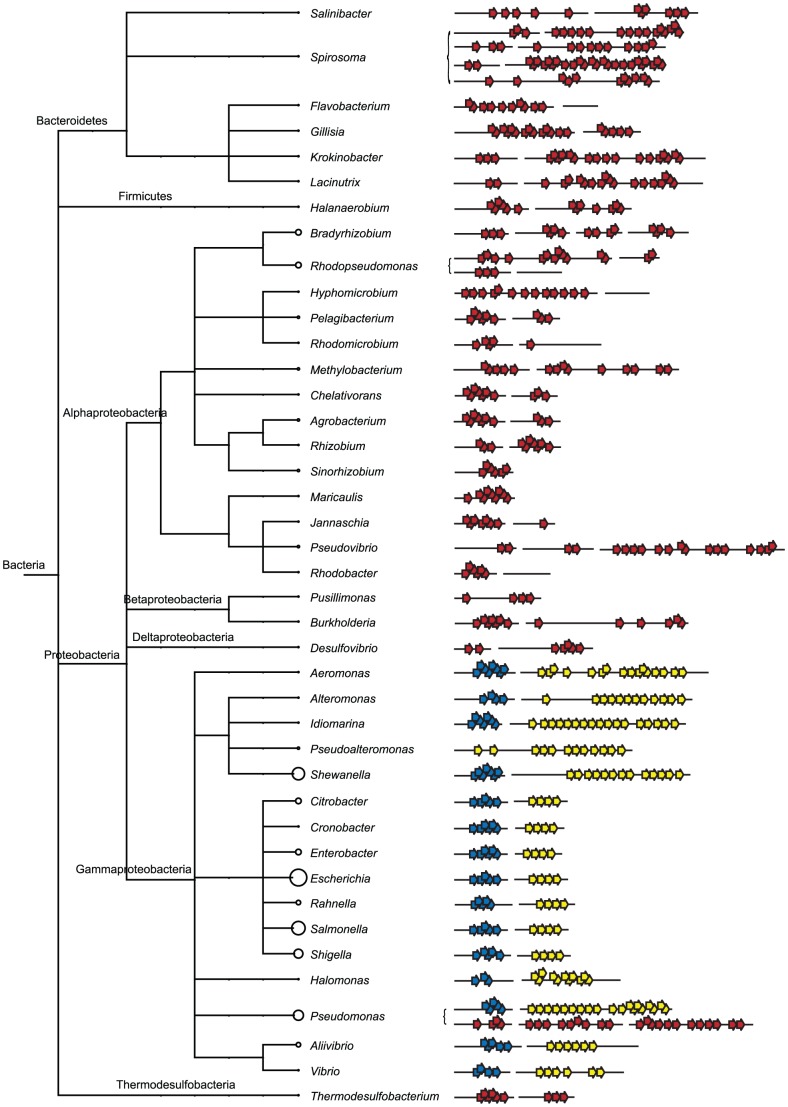
Organization of Curli Repeat Motifs. Minimalistic curli repeats (X_6_QXGX_2_NX_10_) are shown with arrows. Yellow arrows represent repeats within CsgA homologs, blue arrows repeats within CsgB homologs, and red arrows represent repeats in homologs, which cannot be reliably classified as either CsgA or CsgB homologs.

**Figure 3:**
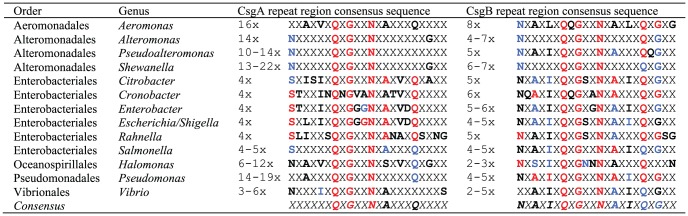
Comparison of Gammaproteobacterial CsgA and CsgB Repeat Regions. Bold residues represent 50% (black), 80% (blue) and 100% (red) conserved residues.

CsgA homologs from different phylogenetic groups show an astonishing variation in size and organization of repeat regions (Figure 2 and Figure 3). Whereas CsgA homologs from the Enterobacteriales are relatively short (∼152 amino acid residues) and contain only 4–5 complete repeat motifs, CsgA homologs from other bacterial orders are up to 529 amino acid residues in length and may contain up to 22 repeat motifs. This variation may have huge impact on polymerization, structure and stability of the resulting FuBA. It is interesting to notice that homologs of the nucleator protein, CsgB, do not show the same variation in size, although this protein is an internal homolog of CsgA (Figure 2 and Figure 3). The repeat motifs are generally well aligned end-to-end inside the CsgA homologs. For some genera, such as *Halomonas*, repeat regions superimpose due to the presence of two minimalistic repeat regions (X_6_QXGX_2_NX_10_) inside the individual repeats. Such overlapping repeats may contribute to an increased stability and rigidity of the resulting FuBA.

Comparison of CsgA and CsgB repeat region consensus sequences from Gammaproteobacteria shows that the CsgB repeat regions are much better conserved then the CsgA repeats (Figure 3). Two minimalistic repeat regions are common for CsgB but not for CsgA. This suggests, that the nucleator function of CsgB impose more structural constraints. There is also significant variation of the repeat region consensus sequence among genera. The highly conserved serine residue of the Enterobacterial CsgA repeats is for example not seen for any other genera.

### Evolution of Curli Systems

**Figure 4:**
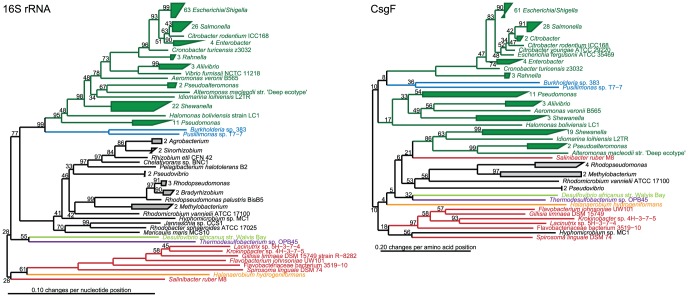
Evolution of Curli Systems. Comparison of phylogenetic trees based on the CsgF protein sequences and corresponding 16S rRNA genes. The trees based on aligned protein and nucleotide data were estimated using distance matrix and maximum likelihood and resulted in congruent tree topologies. Distance matrix trees are shown.

A comparison of phylogenetic trees based on functional genes or protein sequences with those of 16S rRNA gene sequences for the corresponding bacteria can be used to track the evolutionary history and mechanisms of gene transfer of the functional genes. Phylogenetic trees based on the CsgDEFGH proteins sequence and the 16S rRNA gene of the corresponding bacteria show remarkably similar overall topography (Figure 4 and Figures S1, S2, S3, S4). Csg homologs from all genera localized in narrow genus-specific clusters, with the exception of *Shewanella*, which separated into two distant clusters. This denotes that horizontal gene transfer only play a minor role in the spreading of the curli system, and suggests that the curli systems might have evolved from a common ancestor.

### Curli Systems within Metagenomes

**Figure 5:**
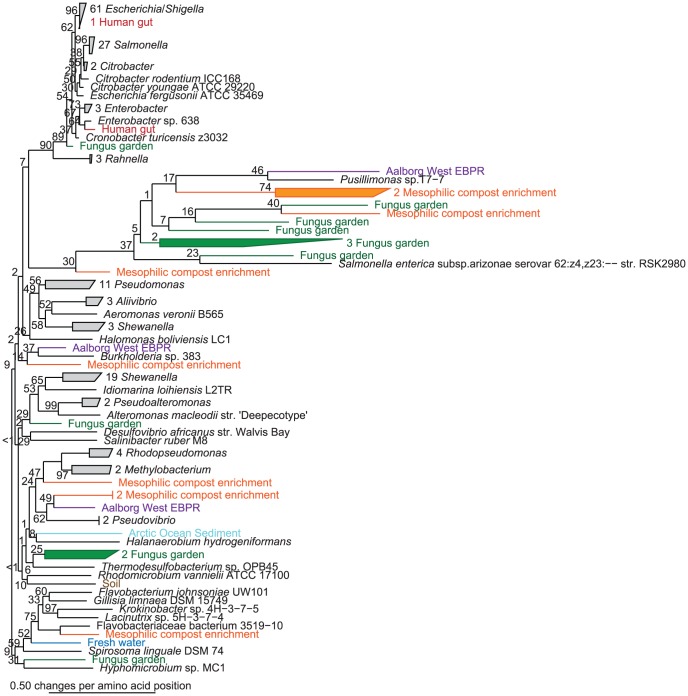
Curli Systems within Metagenomes. CsgF homologs were identified within 10 large metagenomes covering a diverse range of habitats, see Table 2, using the curated CsgF HMM. The hits were aligned with the CsgF homologs identified within refseq and phylogenetic trees were estimated using distance matrix.

Although the number of genome-sequenced bacteria has increased intensively over the last years, there is still a strong bias towards clinically relevant and cultivable bacterial strains. The CsgE, CsgF and CsgH HMMs were therefore used to identify curli homologs within 10 large metagenomes from a diverse range of habitats (Table 2). These HMMs were selected due to their high sensitivity and specificity and because they cover the phylogeny of the curli system well (Table 1). The hits were aligned with the previously identified Csg homologs in order to construct phylogenetic trees (Figure 5 and Figures S5 and S6). All metagenome hits fell within the known phylogenetic diversity of the genome-sequenced bacteria. This indicates that the HMM models based on the genome-sequenced bacteria cover the phylogenetic diversity of the curli system. Some of the metagenome hits formed well-defined clusters in the phylogenetic tree and might therefore represent novel phylogenetic clades. These clusters were mainly seen within the Alpha- and Betaproteobacteria.

**Table 2:** Curli systems within Metagenomes.

Metagenome name	Abbreviation	Size (proteins)	CsgE hits	CsgF hits	CsgH hits	IMG/M taxon object id
Guaymas Basin hydrothermal plume	Hydrothermal plume	319,874	0	0	0	2061766003
Soil microbial communities from sample at FACE site metagenome	Soil	1,057,446	1	1	1	2124908009
Mesophilic rice straw/compost enrichment metagenome	Mesophilic compost enrichment	840,360	2	9	9	2199352012
Thermophilic rice straw/compost enrichment metagenome	Thermophilic compost enrichment	432,661	0	0	0	2199352008
Fresh water microbial communities from LaBonte Lake, Laramie, Wyoming, sample from algal/cyanobacterial bloom material peak-bloom 2	Fresh water	665,401	1	1	0	2189573023
Sediment microbial communities from Arctic Ocean, off the coast from Alaska, sample from low methane PC12-247-20 cm	Artic ocean sediment	784,879	0	1	0	2100351001
Fungus garden microbial communities from Atta colombica in Panama, sample from dump	Fungus garden	1,285,907	6	12	4	2038011000
Svalbard Reindeer rumen metagenome	Reindeer rumen	813,781	0	0	0	2088090000
HumanGut BGI gene set	Human gut	3,064,560	2	2	0	4448044^1^
Aalborg West enhanced biological phosphor removal waste water treatment plant	Aalborg West EBPR	1,636,090	1	3	1	Not published

CsgE, CsgF and CsgH homologs where identified using the developed HMMs.

1 MG-RAST id.

## Discussion

### Curli System are Found in Bacteria from Diverse Habitats

The curli genes are phylogenetically much more widespread than initially appreciated. Curli-like FuBA may consequently be encountered frequently in environmental and pathogenic biofilms and they might have additional functions that are yet to be discovered. It is for example interesting that members of the Rhizobiales contain curli genes. These bacteria form complex symbiotic relationships with legume roots in the form of nitrogen-fixing nodules. As *E. coli* and *Salmonella* curli have been shown to promote binding to plant tissue, curli fimbriae could conceivably play a role in this symbiosis [32], [33]. Besides being found in many soil and enteric bacteria, curli genes were also found within many genera of marine bacteria, including *Alteromonas*, *Pseudoalteromonas*, *Shewanella*, *Halomonas*, and *Vibrio* and *Oceanicola*.

### Curli as a Virulence Factor

Curli fimbriae are known virulence factors in *E. coli* and *Salmonella* pathogenicity [31]. The phylogenetic diversity of the curli system documented in this study arise the question whether this is also the case for other potential pathogens containing curli genes. For example, curli genes were identified in *Aeromonas veronii*, which is an emerging pathogen [34] that may cause diseases ranging from serious wound infections to septicemia in humans [35]. Although they are mostly isolated from cases of gastroenteritis that can range from a mild self-limiting diarrhea to cholera-like illness and dysentery [36], it is still poorly understood how *A. veronii* cause infections, but various adhesins may be involved [34]. Curli-like amyloids might mediate binding to and internalization into host cells as is seen for *E. coli*. Another example is *Cronobacter turicensis*, a food-borne pathogen associated with acute infections in neonates, which may lead to clinical outcomes such as meningitis, sepsis and necrotizing enterocolitis [37]. This bacterium is capable of forming biofilms that may contribute to pathogenicity [38]. An obvious experiment will be to investigate by genetic knock-out methods whether curli-like amyloid contributes to the biofilm development and virulence by *C. turicensis*.

### Alternative Regulation and Biogenesis

Large flexibility is observed in the organization of *csg* genes across both genera and orders. For some genera the genes are divided into two operons, as seen for *E. coli*. In others they form a single operon. Genome rearrangements as seen for the *csg* operons are not uncommon in bacterial evolution [39], [40]. Although operon rearrangement or disruption may result in loss of gene function, this does not have to be the case. The evolution of tryptophan synthesis (*trp*) operons is a clear-cut example of this. Whereas the six *trp* genes have evolved into a single transcriptional unit in *E. coli*, a separation into three transcriptional units is seen for *P. aeruginosa* [41]. Splitting of the *trp* operon does not affect physiological function, but it leads to alternative regulation, as each individual transcriptional unit requires its own transcription factors [41]. The overall variation in *csg* gene organization can therefore not be used to determine operon functionality. It does, however, imply that transcriptional regulation of curli biogenesis may vary between bacteria of different orders. The organization of *csg* into a single operon, as seen for *Pseudoalteromonas*, indicates that all genes are co-transcribed. Regulation of the individual Csg proteins is therefore required to take place during or after translation. For all *Pseudomonas* species except *P. stutzeri*, no *csgD* homologs are found in the vicinity of the *csg* operons. It might therefore be suspected that different transcriptional regulators control the expression of these operons.

For many of the Alphaproteobacteria, Csg homologs could only be identified for the major and minor curlin subunits. This implies that alternative proteins must fulfill the role of the other Csg proteins in order to allow the bacteria to express functional curli fimbriae. This also suggests the existence of different pathways to amyloid biogenesis. Interestingly, the *csg* operon in these Alphaproteobacteria contains an additional gene, which we term *csgH*. The CsgH protein does not have any homologs of known function. A detailed study will therefore be required to determine the role of this protein.

### Variation in the Number of Repeat Units

Whereas CsgA homologs from the Enterobacteriales are relatively short and contain only 4–5 repeat units, homologs from other bacterial orders can be much larger and display up to 22 repeat units (Figure 3). This could have profound effect on the amyloidogenicity of the proteins, the process of CsgA secretion and amyloid formation as well as on the stability and morphology of the resulting fimbriae. The size of CsgG, which constitutes the outer membrane macromolecular exporter, through with the curli subunits are secreted, is unaffected by the huge differences in size of CsgA homologs. This suggests that CsgA and CsgB are secreted as natively unfolded proteins.

Expansion of the oligopeptide repeat domain (ORD) within the mammalian prion protein (PrP) is associated with dominant, inherited prion diseases [42]. The effect of repeat expansion on amyloid formation has been investigated in details using chimeric versions of the yeast prion protein Sup35p, in which the ORD have been replaced by wildtype or expanded ORDs from PrP [43], [44]. *In vitro* studies showed that expansion of repeat regions increase the aggregation propensity and the kinetics of fibril formation, the latter to such a degree that no lag phase can be observed. The repeat expansion showed no detectable effect on fibril stability, suggesting that a similar amyloid core was formed. The repeat expansion furthermore had a clear effect on the morphology of the formed fibrils. The wildtype construct produced fibrils that were long and straight, whereas the expanded construct resulted in curvy and clumped fibrils [44]. The results of the *in vitro* studies were in line with observations previously made on the phenotypic effects of similar mutations in yeast cells [43]. From the prion data we might expect that expansion of repeat units in CsgA homologs results in more aggregation prone proteins, which form amyloids with comparable stability. Mutation of gatekeeper residues, i.e. residues that reduce amyloidogenicity, in the *E. coli* CsgA repeats results in CsgA monomers, which *in vitro* forms amyloid fibrils in the absence of a nucleator [45]. Consequently, it is possible that some species are able to express fimbriae in the absence of a nucleator protein, although this might reduce control of the aggregation process. An alternative could be that gatekeeper residues within individual repeat units are used to dampen the amyloid propensity, thereby allowing the fibrillation to be controlled by CsgB.

### Functional FuBA Systems or Pseudogenes?

The presence of curli gene homologs within the genome of a bacterium does not necessarily imply that the bacterium is able to express functional curli fimbriae. The genes may simply be pseudogenes, which have been passed on through vertical gene transfer. However, theoretical considerations suggest that the curli systems are functional. Although the CsgA homologs have been subjected to many mutation and recombination events, as judged by the low sequence identity between homologs and the large variation in the number of repeats, these do not seem to affect the highly organized modular structure of repeat units lined up within the CsgA homologs. Recombination events sometime occur within the repeats. Yet this does not lead to disruption of the repeat motif. The positive selection for repeat motifs implies that the proteins are functional. In addition, the reorganization of the csg operons seldom results in disrupted genes. Gene disruption should be much more common if the *csg* homologs were pseudogenes as there is no selective pressure acting against their disruption. We therefore suspect that the homologous curli systems are functional.

### Multiple FuBA Operons suggest Multiple FuBA Systems

Curli homolog systems are found within *Pseudomonas putida* F1 and *P. fluorescens* Pf0-1. These strains also contain an operon coding for another type of FuBA, namely the Fap fimbriae [46]. This opens up for the possibility that some bacteria are able to express several FuBA depending on the environmental conditions. These FuBA may have different functions. One could be used for initial adhesion or host interaction, whereas the other could play a structural role in the mature biofilm. This is supported by the fact that *E. coli* curli mainly are expressed during the stationary phase [19], whereas Fap fimbriae in *Pseudomonas* seem to have the highest expression during the exponential growth phase (Dueholm *et al.*, unpublished results).

### Concluding Remarks

This study clearly demonstrates that only the tip of the iceberg have been investigated in respect to curli functional amyloids. The phylogenetic diversity of curli systems implies that these proteinaceous extracellular polymeric substances (EPS) are common in many biofilms and should be considered of equal importance as polysaccharides and extracellular DNA. The variability in operon and amyloid subunit structure suggests that curli systems have evolved to fulfill specific functions for individual species, however pure culture studies are required to elucidate the exact function of each curli system.

## Methods

### Identification of Homologous Curli Systems

Csg homologs were initially identified by PSI-Blast searches (default settings, blosum45 scoring matrix, E-value<1) against the refseq database using Csg proteins from *E. coli* str. K-12 substr. MG1655 as query sequences [47]. The hits were manually curated based on overall proteins structure and gene location relative to that of related *csg* gene homologs. HMMs were made for each of the Csg protein families using hmmbuild of the HMMER 3.0 package and non-redundant versions of the curated Csg protein datasets aligned using ClustalW (gap opening cost (GOC) = 15 and gap extension cost (GEC) = 1). The structural flexibility and low sequence similarity outside the repeat regions of CsgA and CsgB made confident sequence alignment of these proteins impossible. A combined CsgA/B HMM was therefore made in the same way based solely in the repeat regions. The hmmsearch command of the HMMER 3.0 package was used together with the HMMs to search for additional Csg proteins within the refseq database. The hits were curated and included in the Csg homolog database (Table S1) and the expanded datasets were used to generate improved HMMs. This process was repeated until no further homologs could be identified. Identification of Csg homologs within the metagenome databases was done using a similar approach.

### Identity and Curli Repeat Identification

Curli repeats were identified by motif search in CLC DNA workbench 5.7.1 (CLC Bio, Aarhus, Denmark) using a java regular expression of the minimalistic curli repeat (X_6_QXGX_2_NX_10_) described by Chapman *et al.* [31]. All repeat regions from bacteria within the same genus were aligned (ClustalW, GOC = 50 and GEC = 1) in order to determine repeat region consensus sequences.

### Phylogenetic Analysis

16S rRNA gene sequences were obtained for bacterial strains containing homologous curli systems from the NCBI non-redundant nucleotide database or the Silva 16S rRNA database (http://www.arb-silva.de/). The 16S rRNA gene sequences were aligned using the SINA v. 1.2.9 aligner (http://www.arb-silva.de/aligner/) and imported to the ARB software [48]. The aligned 16S rRNA genes were used to calculate phylogenetic trees based on the neighbour-joining and maximum-parsimony methods provided in the software using the default setups. The two methods resulted in trees with similar overall topology. Homolog Csg protein sequences were aligned (ClustalW, GOC = 15 and GEC = 1) and imported into ARB. Phylogenetic trees were similarly calculated based on the neighbour-joining and maximum-parsimony. These trees also showed similar overall topology.

## Supporting Information

HMMs S1 **Curated Hidden Markov Models for Curli Repeats and CsgC-H.**
(ZIP)

Figure S1 **Phylogenetic Tree Based on the CsgD Protein Sequences.** Trees based on aligned protein data were estimated using distance matrix and maximum likelihood and resulted in congruent tree topologies. The distance matrix tree is shown.
(EPS)

Figure S2 **Phylogenetic Tree Based on the CsgE Protein Sequences.** Trees based on aligned protein data were estimated using distance matrix and maximum likelihood and resulted in congruent tree topologies. The distance matrix tree is shown.
(EPS)

Figure S3 **Phylogenetic Tree Based on the CsgG Protein Sequences.** Trees based on aligned protein data were estimated using distance matrix and maximum likelihood and resulted in congruent tree topologies. The distance matrix tree is shown.
(EPS)

Figure S4 **Phylogenetic Tree Based on the CsgH Protein Sequences.** Trees based on aligned protein data were estimated using distance matrix and maximum likelihood and resulted in congruent tree topologies. The distance matrix tree is shown.
(EPS)

Figure S5 **CsgE Homologs within Metagenomes.** CsgE homologs were identified within 10 large metagenomes covering a diverse range of habitats, see Table 2, using the curated CsgE HMM. The hits were aligned with the CsgE homologs identified within refseq and a phylogenetic tree was estimated using distance matrix.
(EPS)

Figure S6 **CsgH Homologs within Metagenomes.** CsgH homologs were identified within 10 large metagenomes covering a diverse range of habitats, see Table 2, using the curated CsgH HMM. The hits were aligned with the CsgH homologs identified within refseq and a phylogenetic tree was estimated using distance matrix.
(EPS)

Table S1 **Csg Protein Homologs Identified within Refseq.** Csg homologs were identified using the curated HMMs and manual examination of the surrounding gene neighborhoods.
(XLSX)

## References

[1] Collinson SK, Emody L, Muller KH, Trust TJ, Kay WW (1991) Purification and characterization of thin, aggregative fimbriae from *Salmonella enteritidis* . J Bacteriol 173: 4773–4781. 1677357 10.1128/jb.173.15.4773-4781.1991 PMC208156

[2] Collinson SK, Clouthier SC, Doran JL, Banser PA, Kay WW (1996) *Salmonella enteritidis agfBAC* operon encoding thin, aggregative fimbriae. J Bacteriol 178: 662–667. 8550497 10.1128/jb.178.3.662-667.1996 PMC177709

[3] Chapman MR, Robinson LS, Pinkner JS, Roth R, Heuser J, et al (2002) Role of *Escherichia coli* curli operons in directing amyloid fiber formation. Science 295: 851–855. 11823641 10.1126/science.1067484 PMC2838482

[4] Dobson CM (2003) Protein folding and misfolding. Nature 426: 884–890. 14685248 10.1038/nature02261

[5] Gebbink MF, Claessen D, Bouma B, Dijkhuizen L, Wosten HA (2005) Amyloids–a functional coat for microorganisms. Nat Rev Microbiol 3: 333–341. 15806095 10.1038/nrmicro1127

[6] Vidal O, Longin R, Prigent-Combaret C, Dorel C, Hooreman M, et al (1998) Isolation of an *Escherichia coli* K-12 mutant strain able to form biofilms on inert surfaces: involvement of a new *ompR* allele that increases curli expression. J Bacteriol 180: 2442–2449. 9573197 10.1128/jb.180.9.2442-2449.1998 PMC107187

[7] Ryu JH, Kim H, Frank JF, Beuchat LR (2004) Attachment and biofilm formation on stainless steel by *Escherichia coli* O157:H7 as affected by curli production. Lett Appl Microbiol 39: 359–362. 15355539 10.1111/j.1472-765X.2004.01591.x

[8] Kikuchi T, Mizunoe Y, Takade A, Naito S, Yoshida S (2005) Curli fibers are required for development of biofilm architecture in *Escherichia coli* K-12 and enhance bacterial adherence to human uroepithelial cells. Microbiol Immunol 49: 875–884. 16172544 10.1111/j.1348-0421.2005.tb03678.x

[9] Saldana Z, Xicohtencatl-Cortes J, Avelino F, Phillips AD, Kaper JB, et al (2009) Synergistic role of curli and cellulose in cell adherence and biofilm formation of attaching and effacing *Escherichia coli* and identification of Fis as a negative regulator of curli. Environ Microbiol 11: 992–1006. 19187284 10.1111/j.1462-2920.2008.01824.x PMC2672964

[10] Olsen A, Jonsson A, Normark S (1989) Fibronectin binding mediated by a novel class of surface organelles on *Escherichia coli* . Nature 338: 652–655. 2649795 10.1038/338652a0

[11] Olsen A, Arnqvist A, Hammar M, Sukupolvi S, Normark S (1993) The RpoS sigma factor relieves H-NS-mediated transcriptional repression of *csgA*, the subunit gene of fibronectin-binding curli in *Escherichia coli* . Mol Microbiol 7: 523–536. 8459772 10.1111/j.1365-2958.1993.tb01143.x

[12] Collinson SK, Doig PC, Doran JL, Clouthier S, Trust TJ, et al (1993) Thin, aggregative fimbriae mediate binding of *Salmonella enteritidis* to fibronectin. J Bacteriol 175: 12–18. 8093237 10.1128/jb.175.1.12-18.1993 PMC196092

[13] Sjöbring U, Pohl G, Olsén A (1994) Plasminogen, absorbed by *Escherichia coli* expressing curli or by *Salmonella enteritidis* expressing thin aggregative fimbriae, can be activated by simultaneously captured tissue-type plasminogen activator (t-PA). Mol Microbiol 14: 443–452. 7885228 10.1111/j.1365-2958.1994.tb02179.x

[14] Nasr A, Olsén A, Sjöbring U, Müller-Esterl W, Björck L (1996) Assembly of human contact phase proteins and release of bradykinin at the surface of curli-expressing *Escherichia coli* . Mol Microbiol 20: 927–935. 8809746 10.1111/j.1365-2958.1996.tb02534.x

[15] Herwald H, Morgelin M, Olsen A, Rhen M, Dahlback B, et al (1998) Activation of the contact-phase system on bacterial surfaces - a clue to serious complications in infectious diseases. Nat Med 4: 298–302. 9500602 10.1038/nm0398-298

[16] Olsen A, Herwald H, Wikstrom M, Persson K, Mattsson E, et al (2002) Identification of two protein-binding and functional regions of curli, a surface organelle and virulence determinant of *Escherichia coli* . J Biol Chem 277: 34568–34572. 12097335 10.1074/jbc.M206353200

[17] Tukel C, Raffatellu M, Humphries AD, Wilson RP, Andrews-Polymenis HL, et al (2005) CsgA is a pathogen-associated molecular pattern of *Salmonella enterica* serotype Typhimurium that is recognized by Toll-like receptor 2. Mol Microbiol 58: 289–304. 16164566 10.1111/j.1365-2958.2005.04825.x

[18] Tukel C, Wilson RP, Nishimori JH, Pezeshki M, Chromy BA, et al (2009) Responses to amyloids of microbial and host origin are mediated through toll-like receptor 2. Cell Host Microbe 6: 45–53. 19616765 10.1016/j.chom.2009.05.020 PMC2745191

[19] Hammar M, Arnqvist A, Bian Z, Olsen A, Normark S (1995) Expression of two *csg* operons is required for production of fibronectin- and Congo red-binding curli polymers in *Escherichia coli* K-12. Mol Microbiol 18: 661–670. 10.1111/j.1365-2958.1995.mmi_18040661.x. 8817489

[20] Taylor JD, Zhou Y, Salgado PS, Patwardhan A, McGuffie M, et al (2011) Atomic resolution insights into curli fiber biogenesis. Structure 19: 1307–1316. 21893289 10.1016/j.str.2011.05.015 PMC3173608

[21] Gibson DL, White AP, Rajotte CM, Kay WW (2007) AgfC and AgfE facilitate extracellular thin aggregative fimbriae synthesis in *Salmonella enteritidis* . Microbiology 153: 1131–1140. 17379722 10.1099/mic.0.2006/000935-0

[22] Robinson LS, Ashman EM, Hultgren SJ, Chapman MR (2006) Secretion of curli fibre subunits is mediated by the outer membrane-localized CsgG protein. Mol Microbiol 59: 870–881. 16420357 10.1111/j.1365-2958.2005.04997.x PMC2838483

[23] Nenninger AA, Robinson LS, Hultgren SJ (2009) Localized and efficient curli nucleation requires the chaperone-like amyloid assembly protein CsgF. Proc Natl Acad Sci U S A 106: 900–905. 19131513 10.1073/pnas.0812143106 PMC2630086

[24] Otzen DE (2011) Assembling good amyloid: Some structures at last. Structure 19: 1207–1209. 21893282 10.1016/j.str.2011.08.005

[25] Nenninger AA, Robinson LS, Hammer ND, Epstein EA, Badtke MP, et al (2011) CsgE is a curli secretion specificity factor that prevents amyloid fibre aggregation. Mol Microbiol 81: 486–499. 21645131 10.1111/j.1365-2958.2011.07706.x PMC3134098

[26] Romling U, Sierralta WD, Eriksson K, Normark S (1998) Multicellular and aggregative behaviour of *Salmonella typhimurium* strains is controlled by mutations in the *agfD* promoter. Mol Microbiol 28: 249–264. 9622351 10.1046/j.1365-2958.1998.00791.x

[27] Zogaj X, Bokranz W, Nimtz M, Romling U (2003) Production of cellulose and curli fimbriae by members of the family Enterobacteriaceae isolated from the human gastrointestinal tract. Infect Immun 71: 4151–4158. 12819107 10.1128/IAI.71.7.4151-4158.2003 PMC162016

[28] Binnewies T, Motro Y, Hallin P, Lund O, Dunn D, et al (2006) Ten years of bacterial genome sequencing: comparative-genomics-based discoveries. Funct Integr Genomics 6: 165–185. 16773396 10.1007/s10142-006-0027-2

[29] Yang S, Valas R, Bourne PE (2009) Evolution studied using protein structure. In: Gu J, Bourne PE, editors. Structural Bioinformatics. Wiley-Blackwell. pp. 561–573.

[30] Madera M, Gough J (2002) A comparison of profile hidden markov model procedures for remote homology detection. Nucleic Acids Res 30: 4321–4328. 12364612 10.1093/nar/gkf544 PMC140544

[31] Barnhart MM, Chapman MR (2006) Curli biogenesis and function. Annu Rev Microbiol 60: 131–147. 16704339 10.1146/annurev.micro.60.080805.142106 PMC2838481

[32] Barak JD, Gorski L, Naraghi-Arani P, Charkowski AO (2005) *Salmonella enterica* virulence genes are required for bacterial attachment to plant tissue. Appl Environ Microbiol 71: 5685–5691. 16204476 10.1128/AEM.71.10.5685-5691.2005 PMC1265987

[33] Torres AG, Jeter C, Langley W, Matthysse AG (2005) Differential binding of *Escherichia coli* O157:H7 to alfalfa, human epithelial cells, and plastic is mediated by a variety of surface structures. Appl Environ Microbiol 71: 8008–8015. 16332780 10.1128/AEM.71.12.8008-8015.2005 PMC1317338

[34] Hadi N, Yang Q, Barnett TC, Tabei SMB, Kirov SM, et al (2012) Bundle-forming pilus locus of *Aeromonas veronii* bv. Sobria. Infect Immun 80: 1351–1360. 22311923 10.1128/IAI.06304-11 PMC3318429

[35] Parker JL, Shaw JG (2011) *Aeromonas* spp. clinical microbiology and disease. J Infect 62: 109–118. 21163298 10.1016/j.jinf.2010.12.003

[36] Thornley JP, Shaw JG, Gryllos IA, Eley A (1997) Virulence properties of clinically significant *Aeromonas* species: evidence for pathogenicity. Rev Med Microbiol 8.

[37] Carranza P, Grunau A, Schneider T, Hartmann I, Lehner A, et al (2010) A gel-free quantitative proteomics approach to investigate temperature adaptation of the food-borne pathogen *Cronobacter turicensis* 3032. Proteomics 10: 3248–3261. 20718006 10.1002/pmic.200900460

[38] Healy B, Cooney S, O'Brien S, Iversen C, Whyte P, et al (2010) *Cronobacter* (*Enterobacter sakazakii*): an opportunistic foodborne pathogen. Foodborne Pathog Dis 7: 339–350. 19958103 10.1089/fpd.2009.0379

[39] Itoh T, Takemoto K, Mori H, Gojobori T (1999) Evolutionary instability of operon structures disclosed by sequence comparisons of complete microbial genomes. Mol Biol Evol 16: 332–346. 10331260 10.1093/oxfordjournals.molbev.a026114

[40] Price MN, Arkin AP, Alm EJ (2006) The life-cycle of operons. PLoS Genet 2: e96. 16789824 10.1371/journal.pgen.0020096 PMC1480536

[41] Xie G, Keyhani NO, Bonner, Jensen RA (2003) Ancient origin of the tryptophan operon and the dynamics of evolutionary change. Microbiol Mol Biol Rev 67: 303–342. 12966138 10.1128/MMBR.67.3.303-342.2003 PMC193870

[42] Wadsworth JD, Hill AF, Beck JA, Collinge J (2003) Molecular and clinical classification of human prion disease. Br Med Bull 66: 241–254. 14522862 10.1093/bmb/66.1.241

[43] Tank EMH, Harris DA, Desai AA, True HL (2007) Prion protein repeat expansion results in increased aggregation and reveals phenotypic variability. Mol Cell Biol 27: 5445–5455. 17548473 10.1128/MCB.02127-06 PMC1952097

[44] Kalastavadi T, True HL (2008) Prion protein insertional mutations increase aggregation propensity but not fiber stability. BMC Biochem 9: 7. 18366654 10.1186/1471-2091-9-7 PMC2276218

[45] Wang X, Zhou Y, Ren J-J, Hammer ND, Chapman MR (2010) Gatekeeper residues in the major curlin subunit modulate bacterial amyloid fiber biogenesis. Proc Natl Acad Sci U S A 107: 163–168. 19966296 10.1073/pnas.0908714107 PMC2806774

[46] Dueholm MS, Petersen SV, Sønderkaer M, Larsen P, Christiansen G, et al (2010) Functional amyloid in *Pseudomonas* . Mol Microbiol 77: 1009–1020. 20572935 10.1111/j.1365-2958.2010.07269.x

[47] Altschul SF, Gish W, Miller W, Myers EW, Lipman DJ (1990) Basic local alignment search tool. J Mol Biol 215: 403–410. 2231712 10.1016/S0022-2836(05)80360-2

[48] Ludwig W, Strunk O, Westram R, Richter L, Meier H, et al (2004) ARB: a software environment for sequence data. Nucleic Acids Res 32: 1363–1371. 14985472 10.1093/nar/gkh293 PMC390282

[49] Huson DH, Mitra S, Ruscheweyh H-J, Weber N, Schuster SC (2011) Integrative analysis of environmental sequences using MEGAN4. Genome Res 21: 1552–1560. 21690186 10.1101/gr.120618.111 PMC3166839

